# The hidden mechanism of vacuum bell therapy: Local fat hypertrophy drives cosmetic outcome in adolescents with pectus excavatum

**DOI:** 10.1016/j.jpra.2025.12.006

**Published:** 2025-12-14

**Authors:** Xiaoyan Feng, Peter Zimmermann, Martin Lacher, Oliver Johannes Deffaa, Nicole Burger, Johanna Pape, Franz Wolfgang Hirsch, Sebastian Krämer, Daniel Gräfe

**Affiliations:** a Department of Pediatric Surgery, University Hospital, Liebigstraße 20a, 04103 Leipzig, Germany; b Department of Pediatric Surgery, Helios HSK, Ludwig-Erhard-Str. 90, 65199 Wiesbaden, Germany; c Department of Pediatric Radiology, University Hospital, Liebigstraße 20a, 04103 Leipzig, Germany; d Division of Thoracic Surgery, University Hospital, Liebigstraße 20, 04103 Leipzig, Germany

**Keywords:** Pectus excavatum, Vacuum bell, Real-time magnetic resonance imaging, Adolescents

## Abstract

**Purpose:**

Vacuum bell (VB) therapy is a non-invasive treatment option for pectus excavatum (PE), traditionally considered most effective in children under 12 due to greater skeletal remodeling potential. However, the extent and mechanisms of cosmetic improvement in older adolescents remain poorly understood.

**Methods:**

We retrospectively analyzed 19 male patients (median age 14.8 years) who underwent VB therapy for at least 1 year. Real-time MRI was performed before and after therapy to assess morphological changes. Key parameters included the Haller Index (HI), Correction Index (CI), and pectus excavation depth (PED). Additionally subcutaneous soft tissue thickness was measured at the deformity site and at the lateral thoracic wall.

**Results:**

After a median treatment duration of 1.8 years, HI and CI did not show significant reductions (HI: 5.4 to 5.3, *p* = 0.40; CI: constant 32 %, *p* = 0.96), while PED decreased significantly (16.7 mm to 112.7 mm, *p* = 0.009). Subcutaneous soft tissue at the deformity site increased markedly by a median of 6.5 mm, compared to a 0.5 mm increase at the lateral thoracic wall. Fat-selective T1-weighted Dixon MRI confirmed that the increased subcutaneous tissue consisted of adipose tissue.

**Conclusions:**

The improvement in visual appearance of PE morphology during VB therapy results primarily from localized adipose tissue hypertrophy, while skeletal remodeling is likely to contribute to a much lesser degree. From a cosmetic perspective, these findings might justify the continued use of VB therapy beyond the traditional age threshold.

## Introduction

Pectus excavatum (PE), the most common congenital chest wall deformity, is characterized by a posterior depression of the sternum. This condition can lead to a range of physical and psychological consequences, including reduced respiratory capacity, exercise intolerance, and diminished self-esteem.^1^ While surgical correction is typically reserved for severe cases, vacuum bell (VB) therapy has emerged as a non-invasive alternative for patients with mild to moderate PE, offering the potential to correct chest wall deformities without the inherent risks of surgery.^2^^,^^3^

Minimally invasive repair of pectus excavatum (MIRPE), using the Pilegaard modification of the Nuss procedure, is generally not performed before the age of 14. Consequently, in this age group, even patients with PE indices exceeding the typical definition of mild to moderate severity were managed non-operatively.^4^ The VB device consists of a silicone cup applied to the anterior chest wall, generating negative pressure to mechanically elevate the sternum. This sustained traction promotes gradual structural remodeling over time.^5^ A long-term retrospective study from the Netherlands, with a follow-up period of 64 months, reported sustained sternal elevation in 52.1 % of adolescents (<18 years), along with notable improvements in exercise tolerance and body image.^6^ Patient age has been identified as a key factor influencing the success of VB therapy. Several studies have demonstrated that initiating treatment before the age of 11 is predictive of excellent outcomes.^7^, ^8^, ^9^ However, more recent evidence suggests that highly compliant patients may achieve favorable results regardless of age or deformity severity.^10^ Despite these findings, the suitability of VB therapy as a first-line treatment for older adolescents with PE remains unclear. Previous studies have shown that free-breathing, real-time MRI is a reliable, rapid, and radiation-free imaging modality for the assessment of pectus excavatum.^11^^,^^12^

Therefore, the aim of our study was to evaluate the efficacy and underlying mechanisms of VB therapy in adolescents based on real-time MRI.

## Methods

### Cohort

This retrospective study was conducted between April 2020 and April 2025 at a tertiary care center. It included patients with pectus excavatum who underwent vacuum bell therapy and were followed in the specialized chest wall deformity outpatient clinic of the Department of Pediatric Surgery of the University Hospital Leipzig. Patients were excluded if they had not undergone MRI both before and at least 1 year after the initiation of vacuum bell therapy. As a result, two MRI scans acquired at different time points were available for each patient. Female patients were also excluded, as the extent of the deformity is difficult to assess due to the surrounding, developing female breast. The local ethics committee granted approval for this retrospective study. This study received no external funding. The work was supported by institutional resources of the University Hospital Leipzig. The authors adhered to the STROBE guidelines.

### Vacuum bell therapy

At our institution, the initiation of vacuum bell therapy for pectus excavatum (PE) is based on defined eligibility criteria. These included a clear preference for non-surgical treatment, or a definitive refusal of surgical correction.

**Figure 1:**
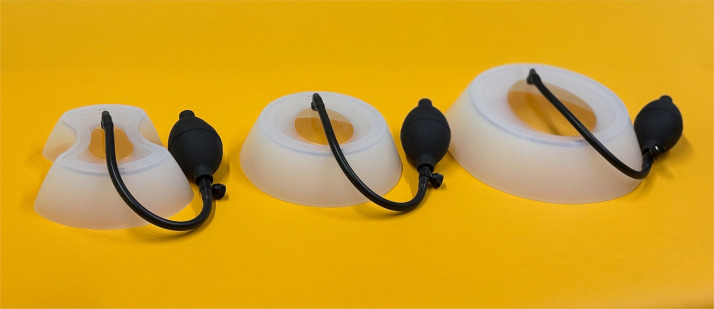
Three vacuum bells for non-surgical therapy for pectus excavatum. Several different sizes for the male and female chest are available.

The selection of the vacuum bell model was individualized based on patient age, thoracic anatomy, and the severity and morphology of the deformity. Three CE-certified standard models (16 cm, 19 cm, and 26 cm in diameter) were available (Figure 1).

The vacuum bell was positioned at the deepest point of the deformity to optimize sternal elevation. Following model selection and initial fitting, patients entered a structured acclimatization phase. This phase consisted of two brief daily application sessions with gradual extension of duration. Within 4–6 weeks, most patients achieved the target regimen of two 30-minute sessions per day. This period facilitated patient habituation and enabled early assessment of treatment response. Patients then transitioned to an intensive treatment phase characterized by daily application times of at least 2 hours. No upper limit for application duration was imposed; rather, patients were encouraged to self-regulate the treatment in alignment with personal tolerance and preferences, consistent with a patient-centered care model.

### Magnetic resonance imaging (MRI)

Thoracic imaging in all patients was performed using real-time MRI. This technique, also known as FLASH2, has been described previously.^11^ In brief, it is based on a highly undersampled, radially acquired gradient echo sequence. A non-linear, iterative reconstruction algorithm estimates the missing image data and coil sensitivity profiles from the previous frame, allowing for temporal resolutions of up to 50 frames per second while maintaining high spatial resolution. This approach minimizes motion artifacts and enables dynamic imaging of the thorax during free breathing.

For the purposes of this study, a transverse sequence was acquired at the level of the deepest point of the pectus deformity during quiet, free breathing, with a temporal resolution of 40 ms. A conventional T1-weighted Dixon sequence was applied for the verification of adipose tissue.

### Measurements

**Figure 2:**
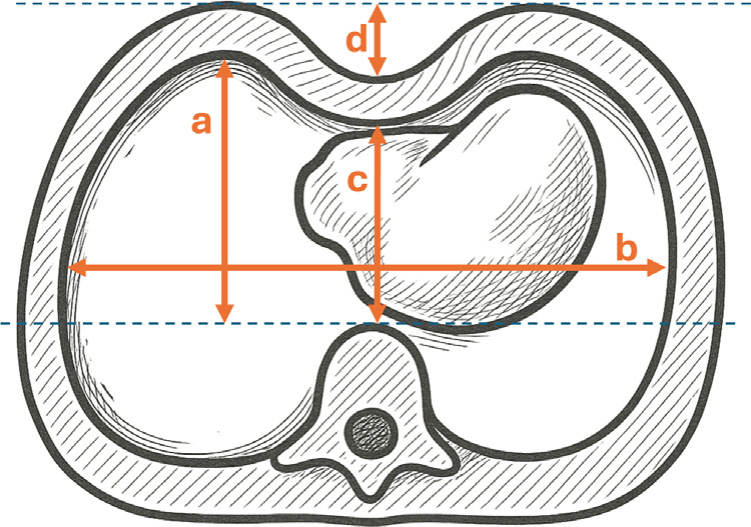
Calculation of chest wall parameters: Haller index = *b*/c; Correction index = (a–c)/a; Pectus excavation depth = *d*. The sashed lines represent auxiliary lines (anterior margin of the spine and anterior surface of the skin).

The Haller Index (HI) and Correction Index (CI) were measured during quiet expiration using established formulas, in accordance with the literature.^11^ The soft tissue thickness was measured in the same plane, perpendicular to the chest wall, at the deepest point of the pectus deformity as well as bilaterally at the ventrolateral thoracic wall, outside the area affected by the vacuum bell. The bilateral lateral measurements were averaged for further analysis. The PED was measured at skin level as a perpendicular line from the deepest point of the deformity to a tangent drawn along the left and right anterior chest wall (Figure 2). All measurements were performed by two readers in independent sessions: a pediatric radiologist with 10 years of experience and a study nurse with 4 years of experience in MRI imaging of pectus excavatum.

### Patient questionnaire

To assess subjective treatment satisfaction, a standardized telephone questionnaire was administered after a minimum of 1 year of VB therapy. The questionnaire consisted of four items addressing: (1) cosmetic result (visible improvement of chest appearance), (2) functional condition (respiratory and physical performance), (3) burden of therapy (pain, effort, limitations), and (4) willingness to repeat the therapy or recommend it to others. Responses were recorded on four-point Likert-type scales.

### Descriptive statistics

Descriptive statistics were performed using RStudio v2024.12.1.563 (PBC, Boston, USA). Measurements from both readers were averaged. Group characteristics were reported as mean with standard deviation or median with interquartile range (IQR), where appropriate. Depending on the distribution of the data, either a paired *t*-test or a Wilcoxon signed-rank test was used to compare pre- and post-intervention measurements. To quantify the effect size of paired mean differences, Cohen’s *d* for dependent samples was calculated. Cohen’s *d* reflects the standardized mean difference and allows interpretation of the practical relevance of changes independently of sample size. Values of *d* = 0.2, 0.5, and 0.8 are typically interpreted as small, medium, and large effects, respectively. A significance level of 0.05 was defined. The study size was determined by the availability of patients meeting inclusion criteria within the study period. No formal sample size calculation was performed due to the exploratory character of this study. Potential sources of bias were minimized by using standardized MRI protocols and independent double reading of measurements by two trained observers. Female patients were excluded to avoid bias from breast tissue development.

## Results

### Patient cohort

Nineteen males were included in the study (median age at the start of vacuum bell therapy: 14.8 years, IQR 14.1–17.3 years, range 9.3–33.2; Table 1). There were no relevant missing data for the main outcome variables (HI, CI, PED, subcutaneous tissue thickness). All patients had complete MRI datasets at both time points. The median percentile of BMI was 19.2 (IQR 18.0–21.8), and the median treatment duration until the second MRI was 1.8 years (IQR 1.5–2.8). No patient required conversion to surgery. The mean HI at baseline was 5.6 ± 1.3.

**Table 1:** Demographics and basic treatment data of the cohort (medians are given).

	Patients
Number (#)	19
Age at start of VB (year)	14.8 (IQR 14.1–17.3)
Duration of VB (year)	1.8 (IQR 1.5–2.8)
BMI percentile	19.2 (IQR 18.0–21.8)

VB, vacuum bell; BMI, body mass index; IQR, interquartile range.

### Objective treatment success

**Figure 3:**
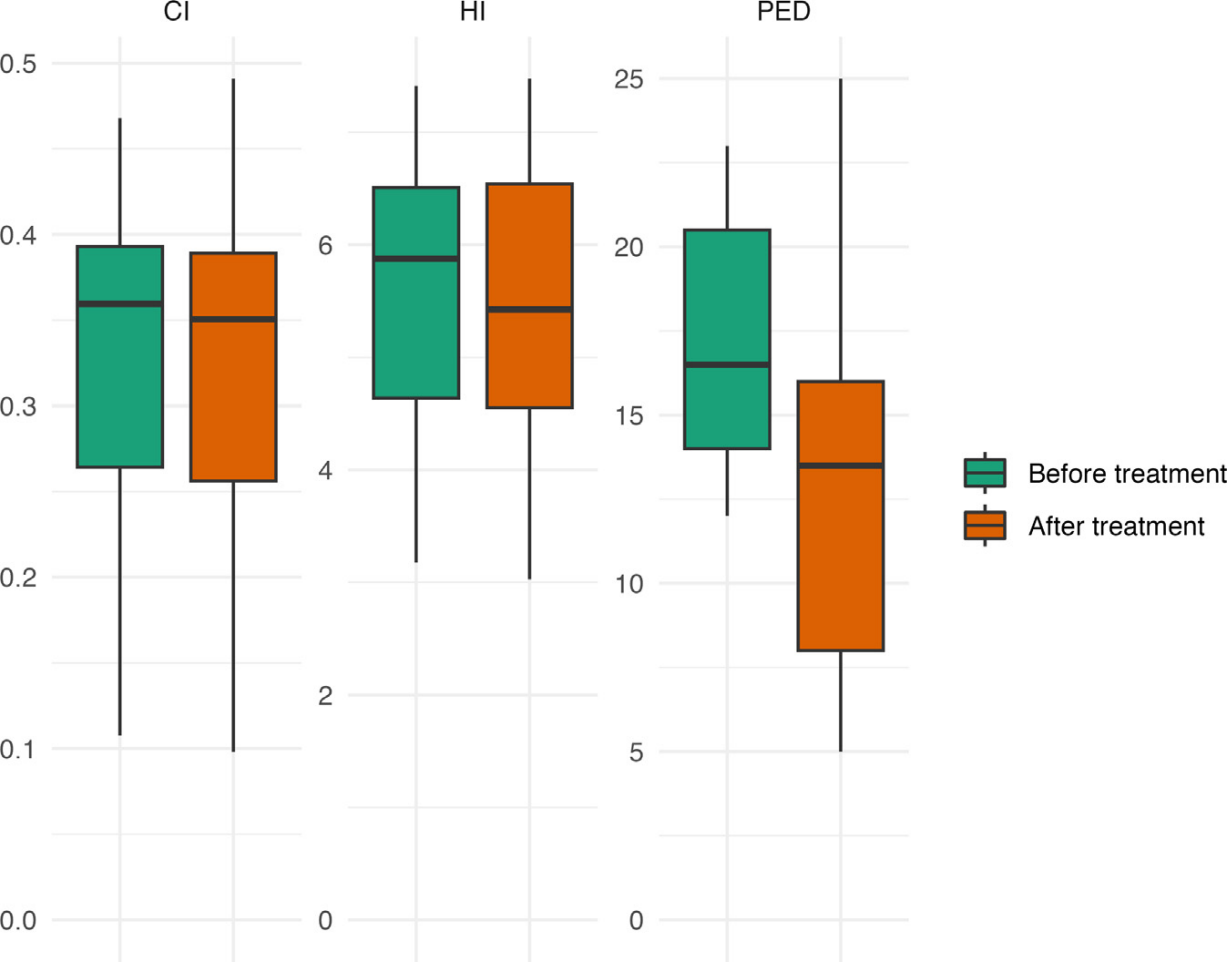
Chest wall indices and depression before therapy (preVB) and at least 1 year after initiation of vacuum bell therapy (postVB) (HI: Haller Index; CI: Correction Index; PED: Pectus Excavation Depth, in mm).

After at least 1 year, VB therapy resulted in reductions in chest wall indices HI and CI, as well as in the depth of the deformity (Table 2 and Figure 3). The reduction in PED reached statistical significance (*p* = 0.009) with a large effect size (Cohen’s *d* = 0.79). In contrast, improvements in HI and CI were non-significant with very small effect sizes (HI: *p* = 0.40, Cohen’s *d* = 0.11; CI: *p* = 0.96, Cohen’s *d* = 0.01). The inter-observer variability was excellent (ICC 0.95 for CI and PED, 0.93 for HI).

**Table 2:** Changes in two chest wall indices and in Pectus excavation depth at skin level before and after at least 1 year of vacuum bell therapy (mean and standard deviation are given).

	Before VB	After VB	*p*-value	Cohen’s *d*
Haller-Index	5.4 ± 1.3	5.3 ± 1.4	0.40	0.11
Correction-index (%)	32 ± 10	32 ± 10	0.96	0.01
Pectus excavation depth (mm)	16.7 ± 4.0	12.7 ± 5.9	0.009	0.79

VB, vacuum bell.

**Figure 4:**
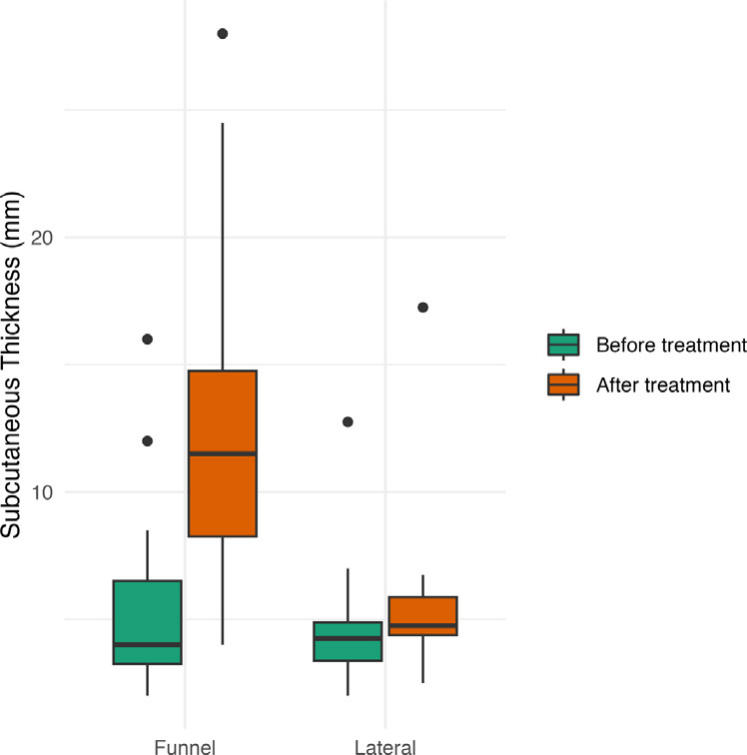
Subcutaneous tissue thickness at the central point of maximal deformity and at the lateral thoracic wall, each measured before and after at least 1 year of vacuum bell (VB) therapy.

**Figure 5:**
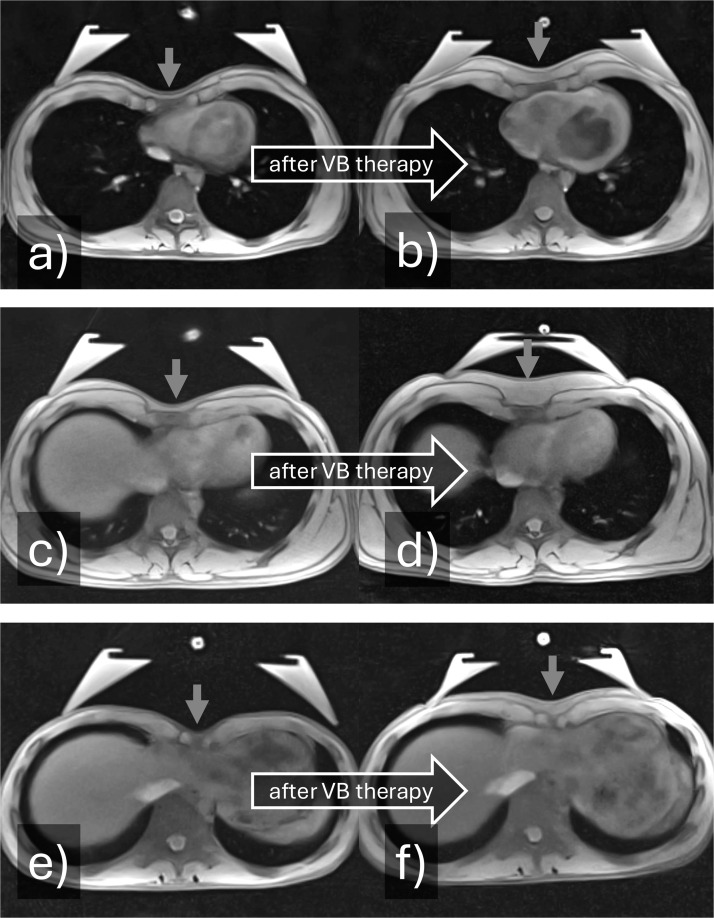
Focal increase of subcutaneous adipose tissue (red arrow) in the funnel after at least on year of vacuum bell (VB) therapy. This represents an interim control, as VB therapy has not yet been completed. Currently the VB is not active. a) 19-year-old boy before and b) after 3 years of therapy; c) 15-year-old boy before and d) after 2 years of therapy; e) 15-year-old boy before and f) after 1 year of therapy.

Subcutaneous soft tissue thickness around the deformity increased during vacuum bell therapy by a median of 6.5 mm (IQR 5.0–8.5 mm). At the lateral thoracic wall, outside the area of vacuum application, the median increase was 0.5 mm (IQR 0.1–1.3 mm) (Figure 4, Figure 5). The interobserver variability was good (ICC 0.78 for the lateral chest wall, 0.86 for the subcutaneous tissue within the area of VB application).

Fat-selective imaging using the T1-weighted Dixon sequence confirmed that the increase in subcutaneous tissue was due to adipose tissue (Supplemental Figure 1).

### Subjective treatment success

**Figure 6:**
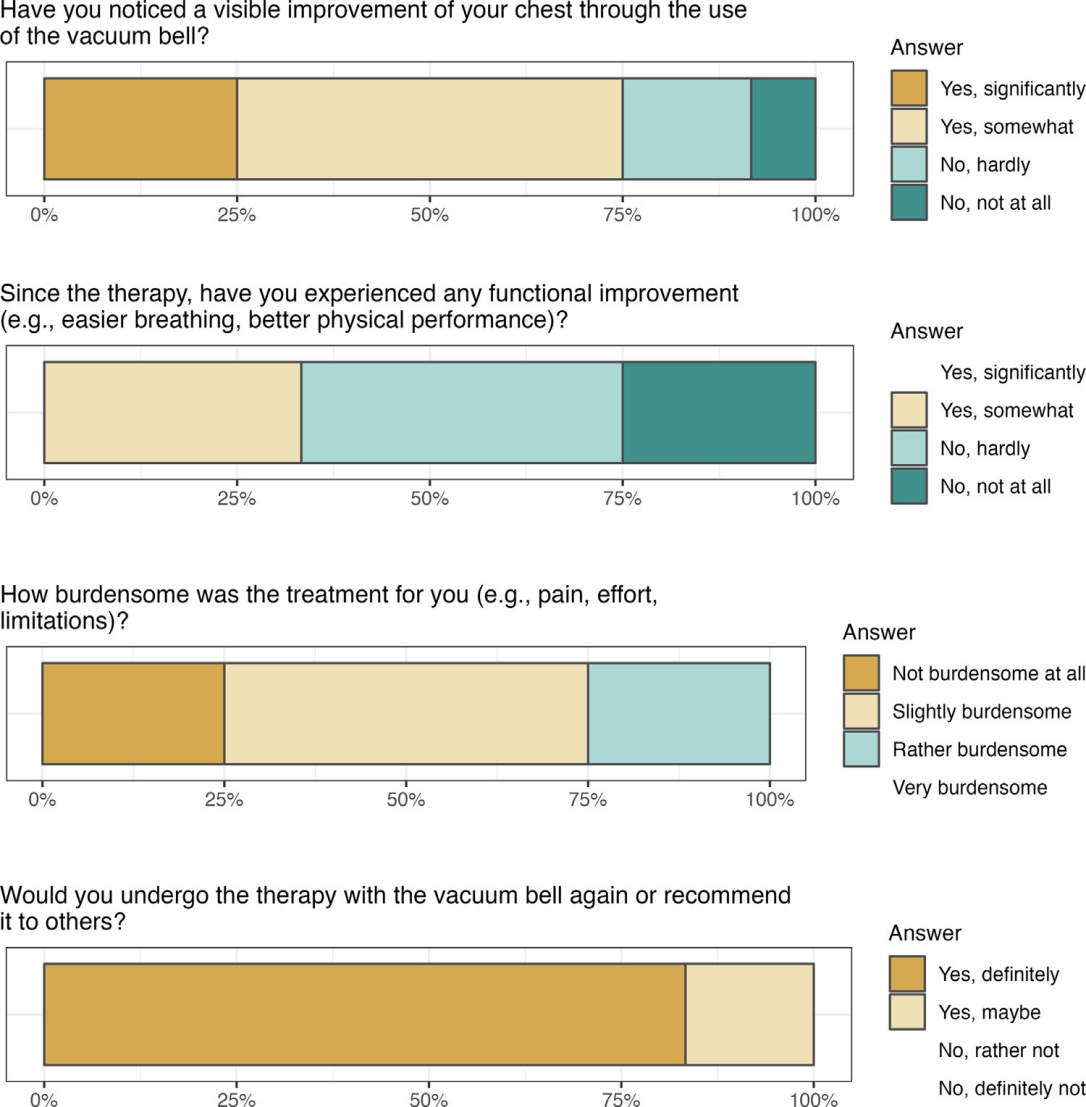
Results of the patient questionnaire on subjective satisfaction after at least 1 year of vacuum bell therapy.

Twelve of nine-teen (63 %) patients completed the questionnaire on subjective satisfaction after at least 1 year of VB therapy. 75 % of patients reported a positive cosmetic effect, whereas only 33 % noted a functional improvement (Figure 6). A total of 75 % perceived the therapy as at most a minor burden. Overall, 83 % of participants indicated that they would definitely undergo VB therapy again, while the remaining 17 % stated they would possibly repeat it. Among the patients treated at our institution—including those enrolled in the study, as well as female patients not included in the analysis and patients who had not yet completed a full year of therapy—none had converted from VB therapy to surgical correction by the time of the study.

## Discussion

This study demonstrates that the apparent clinical improvement in pectus excavatum (PE) morphology during vacuum bell (VB) therapy is not primarily driven by skeletal remodeling, but rather by a pronounced hypertrophy of subcutaneous adipose tissue within the area of the deformity.

VB therapy has gained widespread use as a non-invasive first-line treatment, especially in children and adolescents.^13^^,^^14^ Our findings expand current knowledge by showing that, in older adolescents, cosmetic improvement may result mainly from soft tissue changes. Although the bony indices—HI and CI—showed a trend toward improvement, only the reduction in sternal depression reached statistical significance when comparing pre- and post-treatment. The significant increase in subcutaneous fat thickness under the vacuum bell contributed substantially to this outcome and likely accounts for the improved cosmetic perception reported in previous studies.^10^

Interestingly, the median age at therapy initiation in our cohort was 14.7 years—well beyond the “ideal window” of <12 years, which is generally associated with greater flexibility and better outcomes.^15^, ^16^, ^17^ This observation can be attributed to the fact that, at the beginning of the vacuum bell program at our institution, only adolescent patients presented for evaluation. With increasing awareness of this treatment option—partly due to information sessions for healthcare providers conducted by our team—the age of referred patients gradually decreased. We now regularly treat patients from the age of 10 years.

Despite this, the majority of patients reported aesthetic improvement in the self-report questionnaire, compliance was high, and none opted for surgery. These results support more flexible, individualized treatment criteria, in line with findings from a recent study emphasizing compliance as the key predictor of success.^10^

Although HI and CI improved slightly, these changes did not reach significance. This limited skeletal response in our cohort may be due to age- and maturation-related decline chest wall pliability in older adolescents, unadjusted therapy intensity, and limited sample size. In contrast, real-time MRI revealed significant, localized thickening of subcutaneous fat tissue at the point of maximum depression. Similar findings were reported by Furuta et al., who observed cosmetic improvement in adolescents attributable to subcutaneous changes.^18^ As these alterations were confined to the region under the vacuum bell and largely absent laterally, the findings support a localized effect attributable to the therapy.

The biological basis for this phenomenon remains speculative. However, data from vacuum massage therapy suggest that mechanical stimulation can trigger fibroblast activation, tissue regeneration, and collagen reorganization via nitric oxide signaling and pathways like PKG and ERK1/2.^19^^,^^20^ Another possible biological explaination could be repeated microtrauma under the vacuum bell, leading to minor hematoma and scar formation. This process may resemble the mechanism behind “wrestlers’ ears,” where chronic mechanical stress causes soft tissue thickening through fibrosis and remodeling.^21^ Whether these mechanisms underlie the soft tissue response in VB therapy warrants further investigation.

Importantly, cosmetic perception—not radiological correction—was the main driver for seeking treatment in our cohort. Most patients reported improved body image (own unpublished observations), regardless of bony response. This underscores a paradigm shift: treatment success in PE should not be defined solely by anatomical correction, but also by subjective satisfaction and psychosocial well-being.^22^ Since pectus excavatum correction is pursued not only for cosmetic reasons but also due to suspected cardiopulmonary impairment,^23^^,^^24^ it is problematic to assess treatment success solely based on the externally visible pectus excavatum depth.^6^ As our study demonstrates, PED reflects not only the functionally and cosmetically relevant improvement in chest wall indices such as HI and CI, but also, to a considerable extent, the increase in subcutaneous adipose tissue—which is relevant solely from a cosmetic perspective. Taken together, these findings suggest that vacuum bell therapy is a well-accepted and low-burden treatment option, primarily yielding aesthetic benefits, while functional gains appear to be less consistent. The high level of patient satisfaction despite moderate functional outcomes underlines the importance of cosmetic self-perception as a key determinant of treatment success in patients with pectus excavatum.

VB therapy thus challenges traditional, physician-driven decision models based on age and imaging. Instead, it supports shared decision-making that values patient autonomy, motivation, and perception—especially in adolescent populations.^25^ In this framework, the patient becomes an active participant of treatment success, and subjective body experience gains ethical relevance in clinical decision-making.

The authors acknowledge some limitations of the study: The small sample size limits statistical power and generalizability. The absence of a control group precludes ruling out age-related fat redistribution, although such localized hypertrophy of anterior thoracic fat has neither been reported in the literature nor observed in over 200 rtMRI exams at our institution. Incomplete survey participation could theoretically introduce a bias, as patients dissatisfied with the therapy might be less likely to respond. However, this concern is mitigated by the fact that, to date, no VB patient at our institution has discontinued therapy in favor of surgery. Furthermore, subjective assessments may be biased, and no conclusions can yet be drawn regarding the long-term persistence of the subcutaneous fat tissue growth. To draw more conclusive insights, future studies should include larger cohorts, appropriate control groups, and standardized, validated tools to assess patient satisfaction and cosmetic perception. In addition, molecular studies are warranted to elucidate the underlying biological mechanisms of therapy-induced soft tissue changes.

In conclusion, VB therapy improves chest wall appearance in adolescents to a large extend through localized soft tissue changes, with skeletal remodeling playing a minor role. These findings support the use of the vacuum bell beyond the ideal age - from a cosmetic standpoint. A patient-centered, individualized approach is essential and may better reflect the criteria that define successful treatment of pectus excavatum.

## Funding

None.

## Ethical approval

The study was approved by the local ethics board.

## Declaration of generative AI and AI-assisted technologies in the manuscript preparation process

During the preparation of this work the authors used ChatGPT (OpenAI) in order to spell checking and language smoothing. After using this tool, the authors reviewed and edited the content as needed and take full responsibility for the content of the published article.

## Declaration of competing interest

None declared.

## Appendix Supplementary materials

Image, application 1
