# Supplementary figures and images for: The hidden mechanism of vacuum bell therapy: Local fat hypertrophy drives cosmetic outcome in adolescents with pectus excavatum
Source: JPRAS Open. 2025 Dec 14;48:404–14. doi: 10.1016/j.jpra.2025.12.006 (PMC12803859; doi:10.1016/j.jpra.2025.12.006)

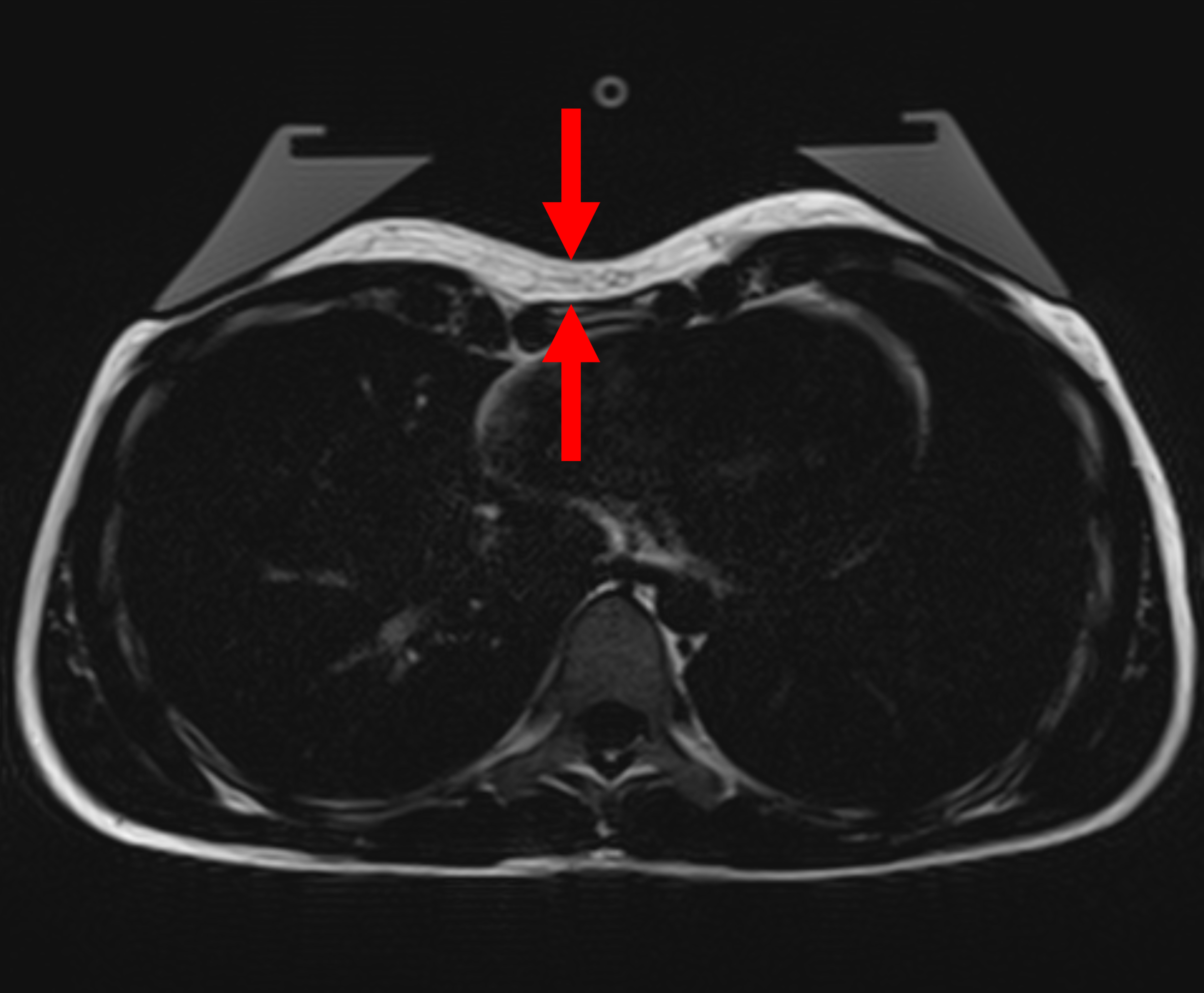

Supplement: Supplementary file 1 [file mmc1.zip › SupplementalFigure1_V2.tif]
